# Supplementary material for: Image Segmentation and Quantification of Droplet dPCR Based on Thermal Bubble Printing Technology
Source: Sensors (Basel). 2022 Sep 23;22(19):7222. doi: 10.3390/s22197222 (PMC9573249; doi:10.3390/s22197222)
Supplement: Supplementary file 1 [file sensors-22-07222-s001.zip › Supplementary File S1.pdf]

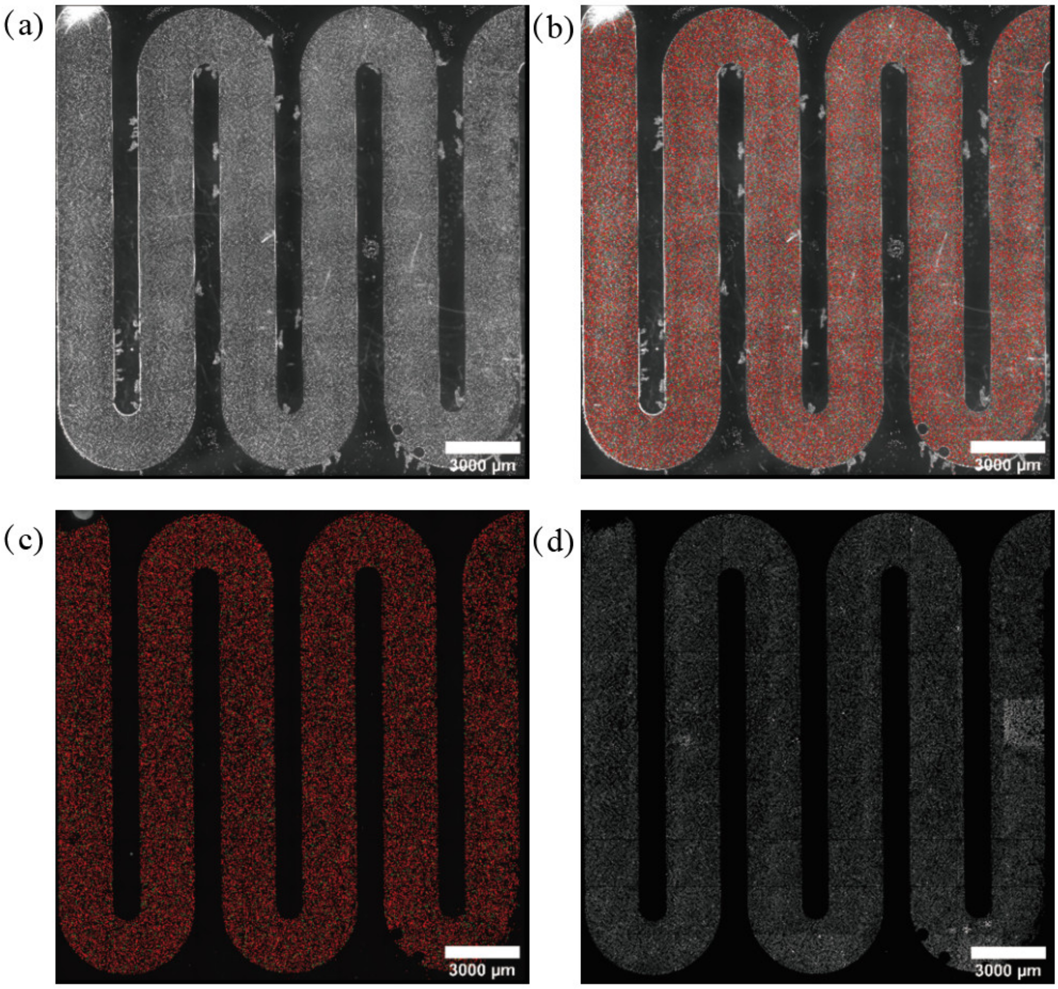

Figure S1 Localization identification of droplets and suppression of the result reading chip contamination result map, each image is stitched from 100 bright-field images or fluorescent images. (**a**) Stitched map of bright-field droplet images captured by the optical module. (**b**) The result of the SimpleBlobDetector algorithm for bright-field image segmentation and localization, screening droplets, and suppressing scratches and bright spots by limiting the area of spots and setting the roundness and convexity of the spots, each red circle represents the outer contour of the identified droplets. (**c**) The resulting graph of suppressing the background impurities of the image. The localization information of the bright field image is used to segment the droplets on the fluorescence field image and calculate the droplet signal value. The droplet signal intensity is higher than the background impurity signal intensity, and setting the threshold value can suppress the background impurity. (**d**) The resulting map of the droplet segmented on the fluorescence channel image using the combined bright-field image positioning information after removing the bright spot of the flow channel sheet and the contamination of the background impurities.

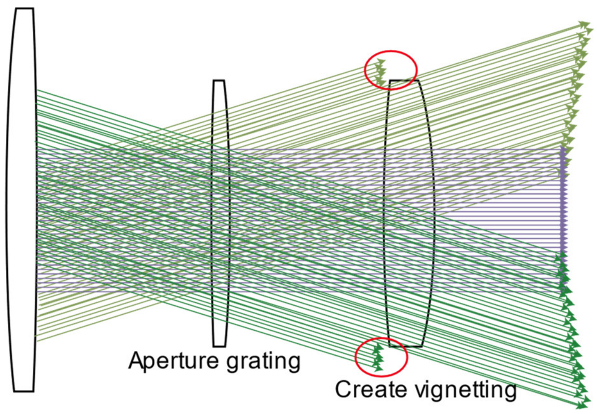

Figure S2. The optical module produces the vignetting phenomenon. Each color of the image represents the light passing through the diaphragm. Due to the limiting effect of the diaphragm and the angle deviation of the light received by the sensor, part of the light beam cannot reach the imaging surface, so the light intensity at the edge of the image decreases, and vignetting occurs.

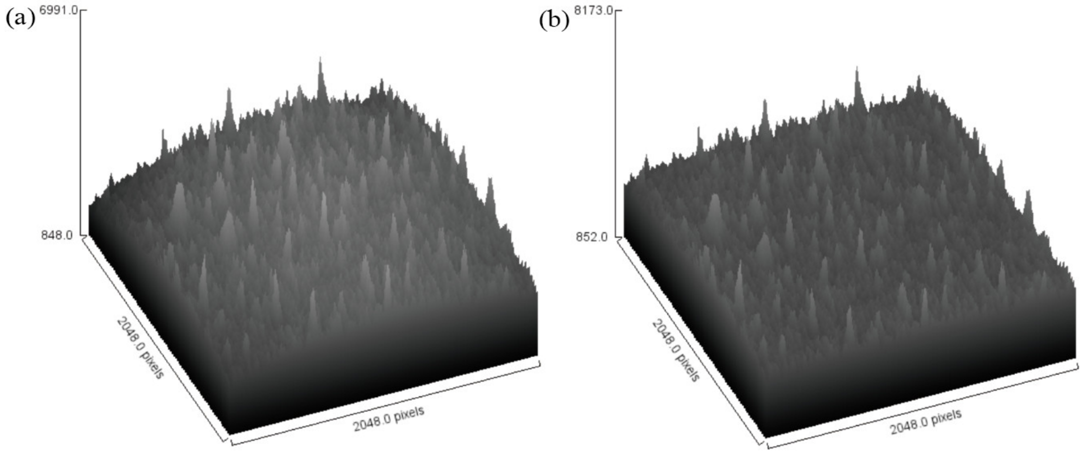

Figure S3. Pixel value surface plots of fluorescence images before and after vignetting correction. (**a**) Pixel-value surface plot of the fluorescence image before vignetting. (**b**) Fluorescence image after vignetting correction.

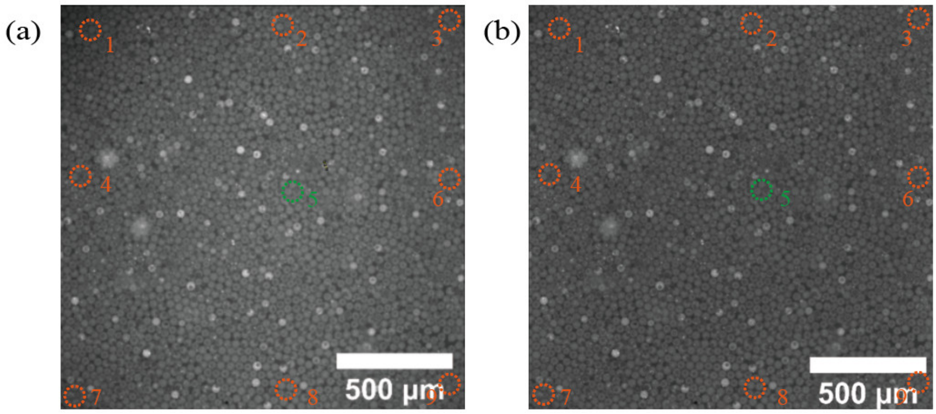

Figure S4. The average gray value of each corner area and center area of the image before and after vignette correction was counted. (a) Statistics before image vignetting correction. (b) Statistics after image vignetting correction.

The droplet generation process may have the situation of small droplets contained in large droplets, and the small droplets contained inside the large droplets are called satellite droplets. The gray value of satellite droplets is about 30% lower than that of negative droplets when imaging, and in addition, bright spots may appear in the negative droplet town and country, and the satellite droplets and bright spots inside the droplets are shown in the attached Figure S5.

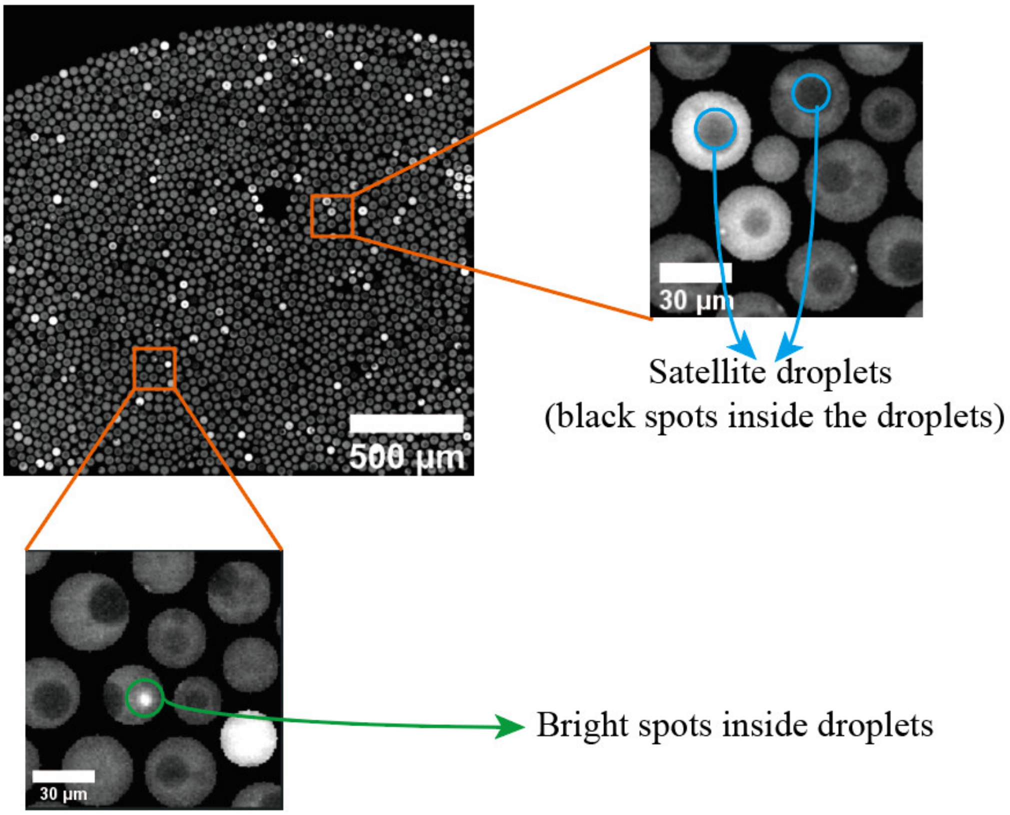

Figure S5. The resulting graph was obtained by segmenting the droplets in the image after vignetting correction of the fluorescence image. The red box droplet magnification results and the arrows indicate the satellite droplets and bright spots inside the droplet.

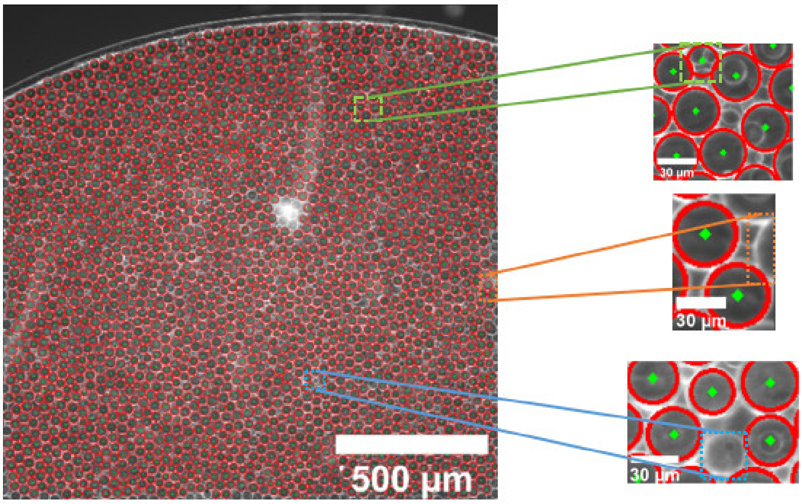

Figure S6. Droplets with wrong segmentation, droplets not validly identified, and droplets with incomplete edges in the image. The green dashed box shows droplets with segmentation errors, the blue dashed box shows droplets that were not validated by the algorithm, and the orange dashed box shows incomplete droplets that were not counted.

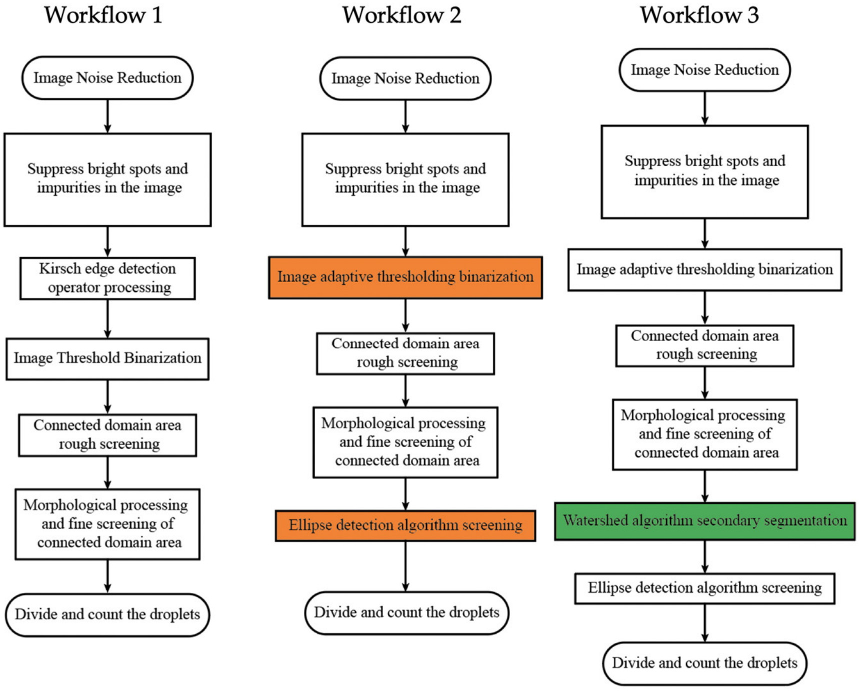

Figure S7. Flow chart of three types of bright field image droplet segmentation workflows. Workflow 1 contains fixed threshold screening, kirsch edge detection operator processing, fixed threshold image binarization, morphology processing, and area screening; Workflow 2 changes fixed threshold image binarization to adaptive threshold binarization screening and adds ellipse detection algorithm screening based on Workflow 1; Workflow 3 adds watershed algorithm for secondary segmentation of images based on Workflow 2, and then uses ellipse detection algorithm for screening.

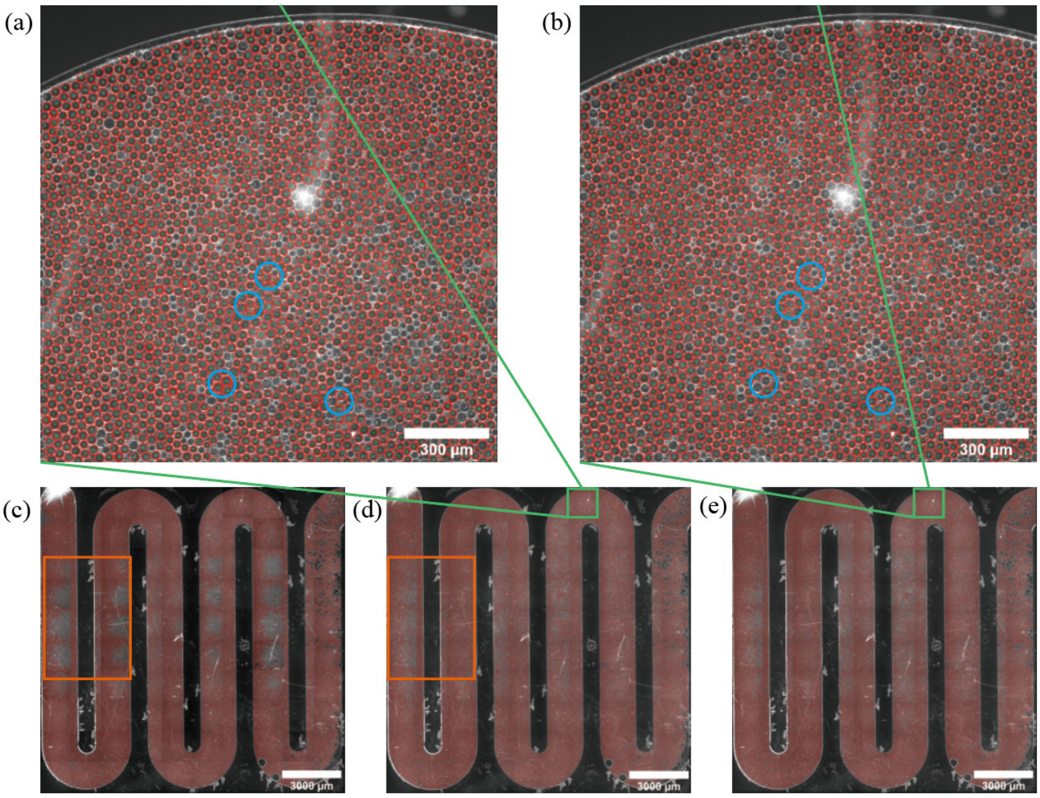

Figure S8. The results of bright-field image droplet segmentation for the three worfkows. (**a**) The result of droplet segmentation for a bright-field image of Workflow 2. The blue circles indicate the mis-segmented droplet areas. (**b**) The droplet segmentation result of a bright-field image of Workflow 3. After the second segmentation using the watershed algorithm, the wrongly segmented region in the blue circle in Workflow 2 on the same image is removed in Workflow 3. (**c**) Stitching map of 100 brightfield droplets segmentations by Workflow 1. The use of fixed thresholds to process the images resulted in some images where the droplets were not segmented well, with the orange boxes showing many regions that failed to be segmented effectively. (**d**) Stitching image of 100 bright-field droplets segmented by Workflow 2. Adaptive thresholding is used to process the images, and the segmentation effect of the images is significantly improved by comparing the orange boxes in Figure S8c,d. (**e**) Stitching image of 100 brightfield droplets segmented by Workflow 3. Workflow 3 adds the watershed algorithm to Workflow 2 for secondary segmentation of the images and then performs elliptical long and short-axis screening.

(a)

Category	CellProfiler	ImageJ	Ilastik	QuPath
% Accuracy	96.2%	92.7%	74.7%	80.9%
% Precision	99.8%	96.3%	80.2%	83.1%

(b) **Our segmentation algorithm is more accurate and stable**

SimpleBlobDetector algorithm

1	1917	1907	1905	2	99.37%
5	8092	8052	8037	15	99.32%
10	17165	17075	17047	28	99.31%
20	33136	33004	32954	50	99.45%

Figure S9. Accuracy values given by different image analysis methods. (**a**) different image analysis methods compared by Sanka I et al. [1], (**b**) the performance of our image analysis method.

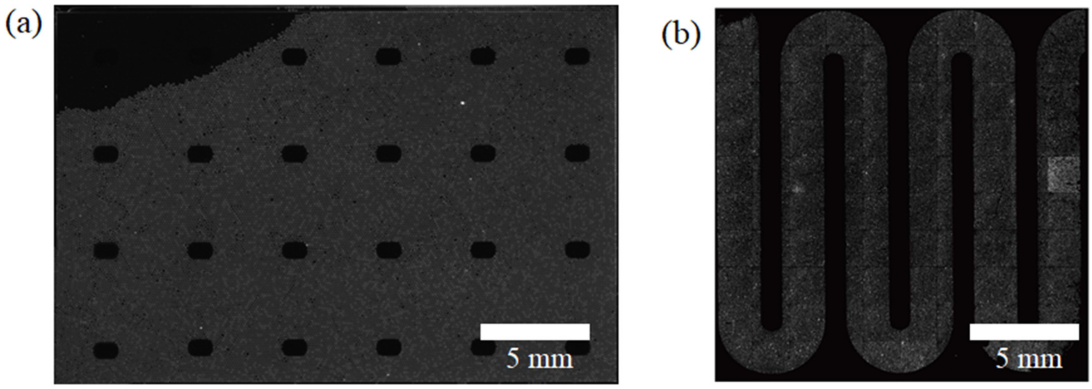

Figure S10. Fill different channels with droplets. (**a**) square channel, (**b**) S shape channel.

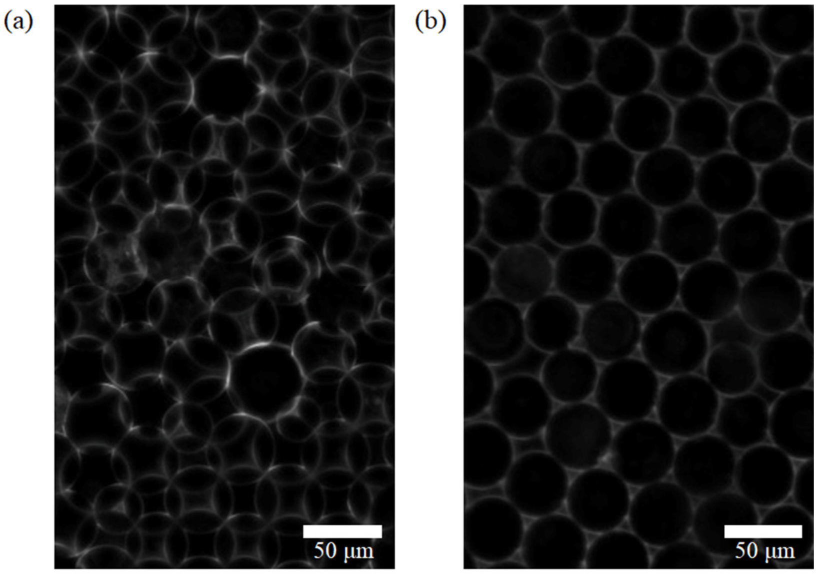

Figure S11. Droplets in multiple layers versus droplets in one layer, (**a**) droplets in multiple layers, (**b**) droplets in one layer.

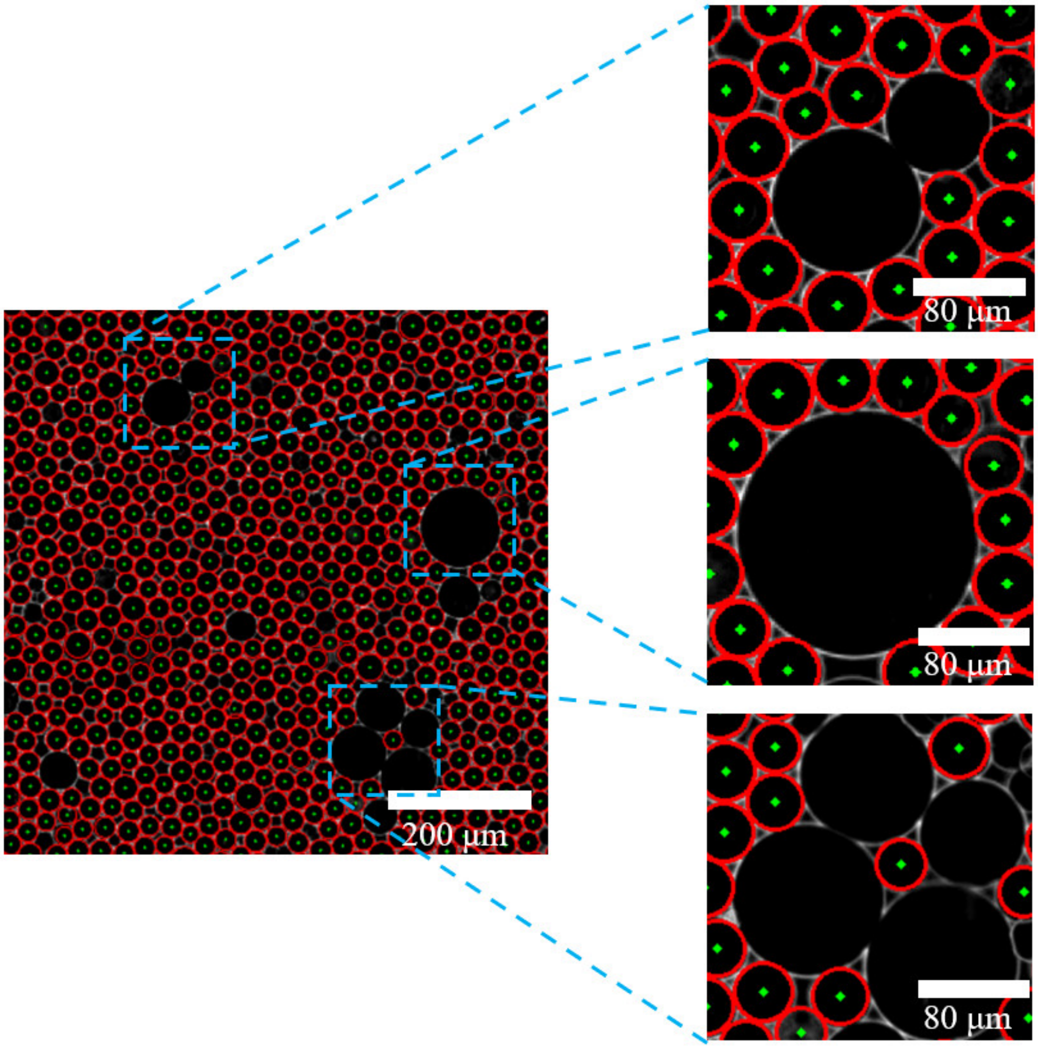

Figure S12. How merged droplets are treated in the image analysis.

Table S1. The difference between the eight corner areas and the central area, and the average of the difference between the eight corner areas and the central area in Figure S4a.

The Serial Number Value of the Orange-Colored Corner Region in Figure S4a	The Average Intensity Of Pixels In Each Orange Corner Region	Average Pixel Intensity in the Green Center Region	Average Pixel Intensity Difference between Each Orange Corner Region and the Central Green Region	Average Of The Difference Between The Average Pixel Value Of The Edge Area To The Average Pixel Value Of The Center Area
1	1950	3371	1421	667
2	3030		341	
3	2667		704	
4	2713		658	
6	2960		411	
7	2498		873	
8	3160		211	
9	2652		719	

Table S2. The difference between the eight corner areas and the central area, and the average of the difference between the eight corner areas and the central area in Figure S4b.

The Serial Number Value of the Orange-Colored Corner Region in Figure S4b	The Average Intensity of Pixels in Each Orange Corner Region	Average Pixel Intensity in the Green Center Region	Average Pixel Intensity Difference between Each Orange Corner Region and the Central Green Region	Average of the Difference between the Average Pixel Value of the Edge Area to the Average Pixel Value of the Center Area
1	2857	3074	217	71
2	3146		72	
3	3064		10	
4	3116		42	
6	3091		17	
7	3003		71	
8	3097		23	
9	3189		115	

1. Sanka, I.; Bartkova, S.; Pata, P.; Smolander, O.-P.; Scheler, O. Investigation of Different Free Image Analysis Software for High-Throughput Droplet Detection. *ACS Omega* **2021**, *6*, 22625–22634. http://doi.org/10.1021/acsomega.1c02664.
